# Spatial heterogeneity in drinking water sources in the Greater Accra Metropolitan Area (GAMA), Ghana

**DOI:** 10.1007/s11111-022-00407-y

**Published:** 2022-08-12

**Authors:** Jacob Doku Tetteh, Michael R. Templeton, Alicia Cavanaugh, Honor Bixby, George Owusu, Sandow Mark Yidana, Simon Moulds, Brian Robinson, Jill Baumgartner, Samuel Kobina Annim, Rosalind Quartey, Samilia E. Mintah, Ayaga Agula Bawah, Raphael E. Arku, Majid Ezzati, Samuel Agyei-Mensah

**Affiliations:** 1 Department of Geography and Resource Development, University of Ghana, P.O. Box LG 59, Legon-Accra, Ghana; 2 Department of Civil and Environmental Engineering, Imperial College London, London, UK; 3 Department of Geography, McGill University, Montreal, Canada; 4 Department of Epidemiology, Biostatistics and Occupational Health, McGill University, Montreal, Canada; 5 Institute of Statistical Social and Economic Research, University of Ghana, Accra, Ghana; 6 Department of Earth Science, University of Ghana, Accra, Ghana; 7 Ghana Statistical Service, Accra, Ghana; 8 Regional Institute for Population Studies, University of Ghana, Accra, Ghana; 9 Department of Environmental Health Sciences, University of Massachusetts Amherst, Amherst, USA; 10 MRC Centre for Environment and Health, School of Public Health, Imperial College London, London, UK

**Keywords:** Drinking water sources, Spatial heterogeneity, Inequality, Census data, GAMA, Ghana

## Abstract

Universal access to safe drinking water is essential to population health and well-being, as recognized in the Sustainable Development Goals (SDG). To develop targeted policies which improve urban access to improved water and ensure equity, there is the need to understand the spatial heterogeneity in drinking water sources and the factors underlying these patterns. Using the Shannon Entropy Index and the Index of Concentration at the Extremes at the enumeration area level, we analyzed census data to examine the spatial heterogeneity in drinking water sources and neighborhood income in the Greater Accra Metropolitan Area (GAMA), the largest urban agglomeration in Ghana. GAMA has been a laboratory for studying urban growth, economic security, and other concomitant socio-environmental and demographic issues in the recent past. The current study adds to this literature by telling a different story about the spatial heterogeneity of GAMA’s water landscape at the enumeration area level. The findings of the study reveal considerable geographical heterogeneity and inequality in drinking water sources not evidenced in previous studies. We conclude that heterogeneity is neither good nor bad in GAMA judging by the dominance of both piped water sources and sachet water (machine-sealed 500-ml plastic bag of drinking water). The lessons from this study can be used to inform the planning of appropriate localized solutions targeted at providing piped water sources in neighborhoods lacking these services and to monitor progress in achieving universal access to improved drinking water as recognized in the SDG 6 and improving population health and well-being.

## Introduction

The past decade has witnessed significant progress in access to drinking water sources globally (Mosello, 2017). Despite these improvements, there are considerable disparities in access to and use of improved water^1^ within cities of the developing world (Alba et al., 2020; Cha et al., 2021; Deshpande et al., 2020; Mutono et al., 2022). Since access to improved drinking water is a central concern for population health and well-being, several international organizations including the United Nations, the World Health Organization (WHO), and United Nations Children’s Fund (UNICEF) have been very proactive in setting targets and providing support for universal access to safe drinking water. For example, the (United Nations, 2015) Sustainable Development Goal (SDG) 6 sets the agenda for addressing inequality in global access to water for all by 2030 with goal 10 aiming at reducing inequalities between and within countries. The 2030 Agenda further commits member states to “leave no one behind” and states that SDG indicators should be disaggregated, where necessary, by income, sex, age, race, ethnicity, migratory status, disability, and geographic location (WHO/UNICEF Joint Water Supply Sanitation Monitoring Programme, 2015). To track progress made with regard to universal coverage, it is essential to understand the geographical variability in drinking water sources and examine the factors driving spatial patterns of drinking water sources and their heterogeneities.

We focus on sub-Saharan Africa (SSA) because a large proportion of the world’s population without access to improved drinking water live in this region (Prins et al., 2022; UNICEF & WHO, 2019), so in a sense SSA is emblematic of the very core of the struggle for improved drinking water. In addition, SSA has the fastest urban population growth; by 2050, SSA’s cities will be home to approximately 950 million more people (OECD/SWAC, 2020). Yet, much of the growth has not been well planned, putting a strain on existing infrastructure and services, especially in emerging settlements (Güneralp et al., 2017). As SSA rapidly urbanizes and improves across economic metrics, demand for access to crucial urban services and infrastructure is growing. Equitable access to quality and affordable housing, clean household energy, efficient transportation, and improved drinking water could improve health, well-being, and productivity in cities (WHO, 2016).

Despite recent attention to household access to improved water and sanitation in resource poor settings, particularly in SSA cities, wide geographical inequities in access to safe, reliable, and affordable water persist within cities (Armah et al., 2018; Deshpande et al., 2020; Hopewell & Graham, 2014; Pullan et al., 2014). While access to urban drinking water in SSA is considered highly heterogeneous (Pullan et al., 2014), within-city water use patterns in SSA cities remain understudied (Adams & Smiley, 2018; Armah et al., 2018; Stoler et al., 2013), presenting a barrier to municipal policy formulation and evaluation.

Previous studies on variations in drinking water sources in SSA have largely focused on between-country analyses (Adams & Smiley, 2018; Armah et al., 2018; Deshpande et al., 2020; Hopewell & Graham, 2014; Pullan et al., 2014). A few studies have also examined disparities in drinking water within countries and major SSA cities (Cha et al., 2021; Cole et al., 2018; Grace et al., 2017; Osei et al., 2015; Songsore & McGranahan, 1998; Thompson et al., 2000). However, only a handful of studies have examined water drinking patterns at localized settings^2^ for fast growing metropolitan cities in SSA (see Alba et al., 2019; Cha et al., 2021; Cole et al., 2018; Thompson et al., 2000), which is needed to better understand inequality in the provision of local infrastructure and essential services.

Ghana has achieved broad economic progress in recent decades which is reflected in improvements in the household environment, including access to improved water and sanitation and cleaner cooking fuels (Arku et al., 2016). Accra, Ghana’s capital, has engulfed its surrounding districts (often referred to as the Greater Accra Metropolitan Area) in an expansion characterized by low-density urban sprawl (Akubia & Bruns, 2019; Owusu, 2013; Owusu & Oteng-Ababio, 2015). As a result, it faces numerous urban development challenges, including inadequate urban drainage system, inadequate housing, poor connecting roads, and high traffic congestion (Cobbinah et al., 2020). In this regard, the provision of improved water sources as one of the priorities of SDG 6 is relevant as it seeks to achieve universal and equitable access to improved drinking water for all by 2030 (Deshpande et al., 2020; United Nations, 2015; UNICEF & WHO, 2019). However, to develop targeted policies which improve urban access to improved drinking water and ensure equity, there is a need to understand the geographical heterogeneity and inequality in drinking water sources.

GAMA is the largest urban agglomeration in the country and accounts for almost a quarter of the national GDP (Gaisie et al., 2019). It has become a laboratory for studying urban growth, resource securities, and other concomitant socio-environmental and demographic issues within the recent past (see Aliu et al., 2021; Bixby et al., 2022; Dapaah & Harris, 2017; Gaisie et al., 2019) and the current study adds to this burgeoning literature by telling a different story about the heterogeneity of GAMA’s water landscape at the enumeration area level. Previous studies relied on sample survey data of selected neighborhoods of GAMA and were not comprehensive enough to identify fine-scale patterns (Ablo & Yekple, 2018; Asante-Wusu & Yeboah, 2020; Benneh et al., 1993; Songsore & McGranahan, 1993; Stoler et al., 2012b). An exception is the case of Moulds et al. (2022) that used census data to partially analyze sachet water consumption among the administrative districts of Ghana but did not examine the spatial heterogeneities of drinking water sources at the enumeration area level.

To analyze the spatial heterogeneity in drinking water sources, we employed the Shannon’s Entropy Index to demonstrate the diversity in sources of drinking water at the enumeration area level. We also used the Index of Concentration at the Extremes to examine neighborhood wealth status. Characterizing spatial heterogeneity of drinking water sources is important as it provides insights into the varied sources of drinking water along with the drivers of water consumption. Additionally, it helps us to know the different strategies people use in their water consumption as well as identify priority areas for intervention. Spatial heterogeneity is good if it means that residents of EAs can find improved drinking water which is publicly managed and affordable. Heterogeneity is bad if it means that residents of EAs are not consistently finding improved drinking water or only finding the more expensive types of improved drinking water, for example, vendor provided.

We specifically address two main research questions: (1) How much heterogeneity in drinking water sources exists within neighborhoods in GAMA?; and (2) does heterogeneity in drinking water source type differ by neighborhood wealth status? The findings of the study can be used to inform the planning of appropriate localized solutions and monitor progress in achieving universal access to safe water as recognized in the Sustainable Development Goal 6 and improving population health and well-being.

## Urban water access in Africa

The research questions posed in this paper intersect with several established fields of research that provide an empirical and theoretical context for studying spatial heterogeneities in water sources. We first briefly consider broad perspectives on sources of urban drinking water. Next, we examine the factors which drive heterogeneity and inequality**.**

### Sources of drinking water

To begin with, there are three broad perspectives on how best to deliver safe drinking water in urban areas in the developing world. The first examines the expansion of piped water infrastructure facilitating the chlorination and filtration of water prior to its distribution (Burrows, 2019). The second view emphasizes individual and household-level interventions such as chlorine-based disinfectants, filtration, and solar disinfection that can be done cheaply and evaluated easily (Geremew & Damtew, 2020; WHO, 2019). The third, and most recent, highlights the growth of a large private sector that distils and distributes water mostly via sachets, bottles, and dispensers (Burrows, 2019; Moulds et al., 2022; Prasetiawan et al., 2017; Zhen et al., 2019).

Urban water access in SSA cities is heterogeneous**.** Planned and affluent residential areas generally have access to piped water services, whereas low-income and peri-urban areas often lack access to the piped network and largely depend on non-piped water services such as sachet water, tanker services, and bottled water (Cole et al., 2018; Deshpande et al., 2020; Geremew & Damtew, 2020; WHO, 2019). However, even where piped water is available, the use of non-piped sources for drinking water is commonplace (Moulds et al., 2022). These broad categories can further be divided into a multiplicity of water sources as provided by the Joint Monitoring Programme (JMP) of the WHO and the UNICEF in the JMP ladder for drinking water services. This new ladder defines 5 service levels. Improved sources are associated with the safely managed, basic, or limited drinking water services^3^ while the unimproved and surface water service levels^4^ are categorized as unimproved (UNICEF & WHO, 2019, see also Table 1). As shown in Table 1, most of the water source types in GAMA are improved sources.

**Table 1:** Classification of drinking water source types in GAMA

Household drinking water facility type in the Census module questionnaire	Number of households	Households coverage (in %)	Grouped coverage (in %)	Classification/grouping	Improved/unimproved systems
Pipe-borne inside dwelling	266,341	27.2	64.2	Piped water sources	Improved
Pipe-borne outside dwelling	276,048	28.2			
Public tap/standpipe	86,669	8.8			
Bottled water	10,728	1.1	32.8	Vendor sources	Improved
Sachet water	283,291	28.9			
Tanker supply/vendor provided	27,120	2.8			
Borehole/pump/tube well	14,891	1.5	2.5	Other sources	Improved
Protected well	4723	0.5			
Rain water	1533	0.2			
Protected spring	3380	0.3			
Unprotected well	1098	0.1	0.5	Other sources	Unimproved
Unprotected spring	218	0.0			
River/stream	2162	0.2			
Dugout/pond/lake/dam/canal	992	0.1			
Other	933	0.1			

### Factors influencing water source heterogeneity and inequality

The review of literature that follows provides the setting for explaining why heterogeneity in drinking water sources exists. Our goal is not to measure these factors in the analysis, but rather to use them to explain the drivers behind these heterogeneities. We hope that future research could incorporate them more explicitly into an explanatory framework for water source heterogeneity.

#### Colonial and post-colonial imprint on the urban landscape

The legacies of uneven development during the colonial and post-colonial period are critical in understanding inequalities in water access in urban areas in SSA. One of the central characteristics of colonial urbanization was residential and spatial segregation perpetuated by unjust land policies, resulting in inequalities in water infrastructure in a number of African cities (Bohman, 2012; Dill & Crow, 2014; Njoh & Akiwumi, 2011; Peloso et al., 2018; Songsore et al., 2009; Tempelhoff, 2003, 2017). Myers (2003) gives an account of how unequal power influenced the production of space under colonial rule in the cities of Nairobi, Lusaka, Zanzibar, and Lilongwe. Njoh and Akiwumi (2011) note that access to improved water supply in East African cities was a function of the duration of the colonial era: access was greater in cities within countries that experienced longer periods of colonization than those in which the colonial era was brief.

Colonial policies have also had a profound impact in shaping the spatial structure of Accra (Andersson, 2017; Brand, 1972; Harris, 2021; Songsore et al., 2009). Harris (2021) notes that the variegated nature of water access in Accra can be traced to the legacies of infrastructure and development during the colonial period that served to condition uneven infrastructure and water flows. Based on field work in Accra, Andersson (2017) observed that the city of Accra is an example of how “segregation works in cyclical, self-reinforcing patterns,” supporting the assertion that segregation and inequality that existed because of colonial policy is an important part in explaining the contemporary socio-spatial structure. Similar segregation policies existed in other colonial West African towns such as Freetown and Dakar (Bigon, 2012; Phillips, 2002).

There is also a growing body of literature on how water governance processes since the post-colonial neoliberal regime of water privatization and the failure of the state to provide sufficient water to the population have led to the presence of new water delivery regimes in GAMA including sachet and bottled water, water tankers, and water vendors (see Alba et al., 2019; Asante-Wusu & Yeboah, 2020; Bartels et al., 2018; Tutu & Stoler, 2016; Yeboah, 2006). It is also interesting to note that since independence in 1957, the residents of the city of Accra have been instrumental in the water network’s granular extension into neighborhoods and across different strata. Thus, what we see today reflects the processes of integration and fragmentation of the water network (Uitermark & Tieleman, 2021). Thus, GAMA is characterized by a multiplicity of water sources including in house piping, private standpipe, communal standpipe, sachet water, bottled water, and water from vendor.

#### Provision of water infrastructure

Closely related to the colonial and post-colonial imprint on the landscape is the issue of the provision of water infrastructure. Recent research in Lilongwe, Malawi, shows that investments in the production of extra water resources mostly benefited those who were already better served (Tiwale et al., 2018). Thus, pipes are not just conduits of water but also of power; infrastructural developments are shaped by economic, social, and political forces that co-determine socio-ecological inequalities (Tiwale et al., 2018). Since the establishment of Lilongwe as a planned city in Malawi, networked infrastructure has grown inequitably over space and time, favoring newly planned central and northern zones of the city that include parliament, ministries, embassies, government offices, hotels, commercial and industrial areas, and the airport while neglecting the low-income areas growing along the southern part of the city (Tiwale, 2019).

In the Accra Metropolitan Area of Ghana, having water connection at home does not necessarily guarantee regular supply because of water rationing and intermittent water flow (Peloso et al., 2018; Stoler et al., 2012a). Because of the uneven water supply outlets within the city, multiple water provision systems have emerged such as tankers, sachet water, and boreholes with a plurality of actors whose practices are informed by a range of motives. These motives go beyond profit-making, political legitimacy, patronage, and petty corruption to include solidarity, religious beliefs, and pragmatic choices (Alba et al., 2020). Maintenance issues with respect to water infrastructure have also restricted water access. In Lusaka, Zambia, the urban water infrastructure built during the 1960s and 1970s has not undergone any major expansion and is insufficient to meet the needs of the current population (Hubbard et al., 2020). A World Bank report published in 2017 also noted the problem of aging infrastructure in the water sector and its accompanying effects, such as leakage of water pipes, and called for more investment in that sector (Van den Berg & Danilenko, 2017). Lack of piped water system maintenance has also been reported by Thompson et al. (2000) for some cities in East Africa. In some instances, the lack of, or limited access to, water in informal low-income and slum neighborhoods is largely due to deliberate policy of city authorities. Where city authorities view emerging slums or poor communities as occupying illegal lands or squatting, they are unlikely to make any infrastructure and service investments in these areas, despite continuous population growth and pleas for these investments by residents (Awumbila et al., 2014; Sinharoy et al., 2019).

There are also issues of trust and human rights when it comes to the provision of water infrastructure. Whereas in Cape Town water quality and satisfaction are linked to trust in government, this is not the case in parts of Accra. For residents of Nima in Accra, water access and quality are important for people’s lives, but are less strongly connected to a sense of governmental responsibility (Harris, 2021). Similarly, in South Africa, water privatization signaled an immense conflict because the country’s constitution indicated that everyone has a right to have access but privatization excluded many poor black South Africans. This is not the case in Ghana where there is a general acceptance of a need to pay for water delivery service (Yates & Harris, 2018).

Dapaah and Harris (2017) provide an entitlement approach to water access that broadens the perspective beyond infrastructural endowments (e.g., piped water), to include a range of other socioeconomic, socio-cultural, and local institutional characteristics. They note in their study in two communities in Accra, Ghana, that among other factors that are important to everyday negotiations and entitlements related to water access are familial and kin networks, water storing options available to households and vendors, the distance and waiting time to fetch water, and local leaders’ perceptions of water issues, particularly how these compare with broader citizen understandings.

Private sector participation in urban water provision and management in many parts of Africa has increased significantly to address the deficit in piped water access. Thus, the consumption of sachet and bottled water has become very common among many households in Ghana, Nigeria, and Sierra Leone (Dada, 2009; Fisher et al., 2015; Stoler et al., 2013). The increasing patronage of sachet water in particular despite some of the issues raised about its quality (see Dzodzomenyo et al., 2018; Mosi et al., 2019) reflects both the failures of municipal water management as well as a lack of funding to extend water services to deprived areas (Stoler, 2017; Stoler et al., 2012b, 2013; Yeboah, 2006). There is also a growing body of literature on how water governance processes such as the role of multiple private water vendors and tanker drivers shape the distribution and access to water especially in peri-urban and low income neighborhoods of GAMA (Alba et al., 2019; Bartels et al., 2018; Tutu & Stoler, 2016).

#### Population growth and urban expansion

In many parts of Sub-Saharan Africa, population growth and urban territory expansion make it difficult for the state and municipal authorities to provide water infrastructure in pace with the increasing needs of the population (Asante-Wusu & Yeboah, 2020; Ayeni, 2017; Cobbinah et al., 2019; Stoler et al., 2013). This is because the distribution systems of network pipes have not changed in any remarkable way since the early independence era. Between 1975 and 2010, the urban population in the Global South tripled, growing by 2 billion people. Most of this growth occurred in Sub‐Saharan Africa and Asia where water insecurity, or the lack of access to adequate and safe water for a healthy and productive life, was already shaping lives and inhibiting development (Adams, 2018). Many of the worst affected areas are slums and informal settlements and areas of intensified growth (Adams, 2018; Angoua et al., 2018; Dos Santos et al., 2017). Thus, over the past decade, a great deal of research has emerged seeking to better understand population growth impacts on water access in Africa (Aliu et al., 2021; Ayeni, 2017; Cobbinah et al., 2019, 2020; Dominguez Torres, 2012; Dos Santos et al., 2017; Hopewell & Graham, 2014; Stoler et al., 2013). Expansion of water service delivery has not happened alongside rapid urbanization. In particular, there is a disconnect between water service delivery and urbanization in Ghana. Water strategies and investments have remained sector-specific and have occurred outside of broader considerations related to urban expansion and the need to serve the rapidly expanding informal and peri-urban settlements (Mosello, 2017).

#### Financial costs

Financial costs are paramount when it comes to understanding variations in water use in urban Africa. The cost of water and the cost of installing water infrastructure have risen due to privatization of water services. Thus, extending service infrastructure to low-income peri-urban areas and newly developed housing schemes is expensive and often technically difficult (Dos Santos et al., 2017). Moreover, having access to a pipe in the home or compound does not guarantee water delivery or adequate piped water quality. Paying for water can be challenging for many residents, as documented in cities in Malawi, South Africa, Tanzania, and Namibia (see Adams & Smiley, 2018; Cole et al., 2018; Mitlin & Walnycki, 2016). In the Greater Accra region of Ghana, the main utility water company, Ghana Water Company Limited (GWCL), is finding it increasingly difficult to keep its customers current with their water bills and provide adequate service because of poor cost recovery issues relating to untimely bill payment from the customers (Sualihu et al., 2017). Studies conducted in Nima, an urban informal settlement in Accra, show that residents are concerned about the quality of water, connection fees, and monthly water bills. The findings provide valuable information that policymakers and water utilities can use to assess the feasibility and cost effectiveness of extending household taps to poor urban settlements (Adams & Vásquez, 2019).

The research presented here explores the diversity in drinking water sources within the various enumeration areas of GAMA. We examine the association between these multiple drinking water sources and income inequalities along with the factors driving these diversity, with the goal of identifying priority areas for policy intervention.

## Method

### Study area

**Fig. 1:**
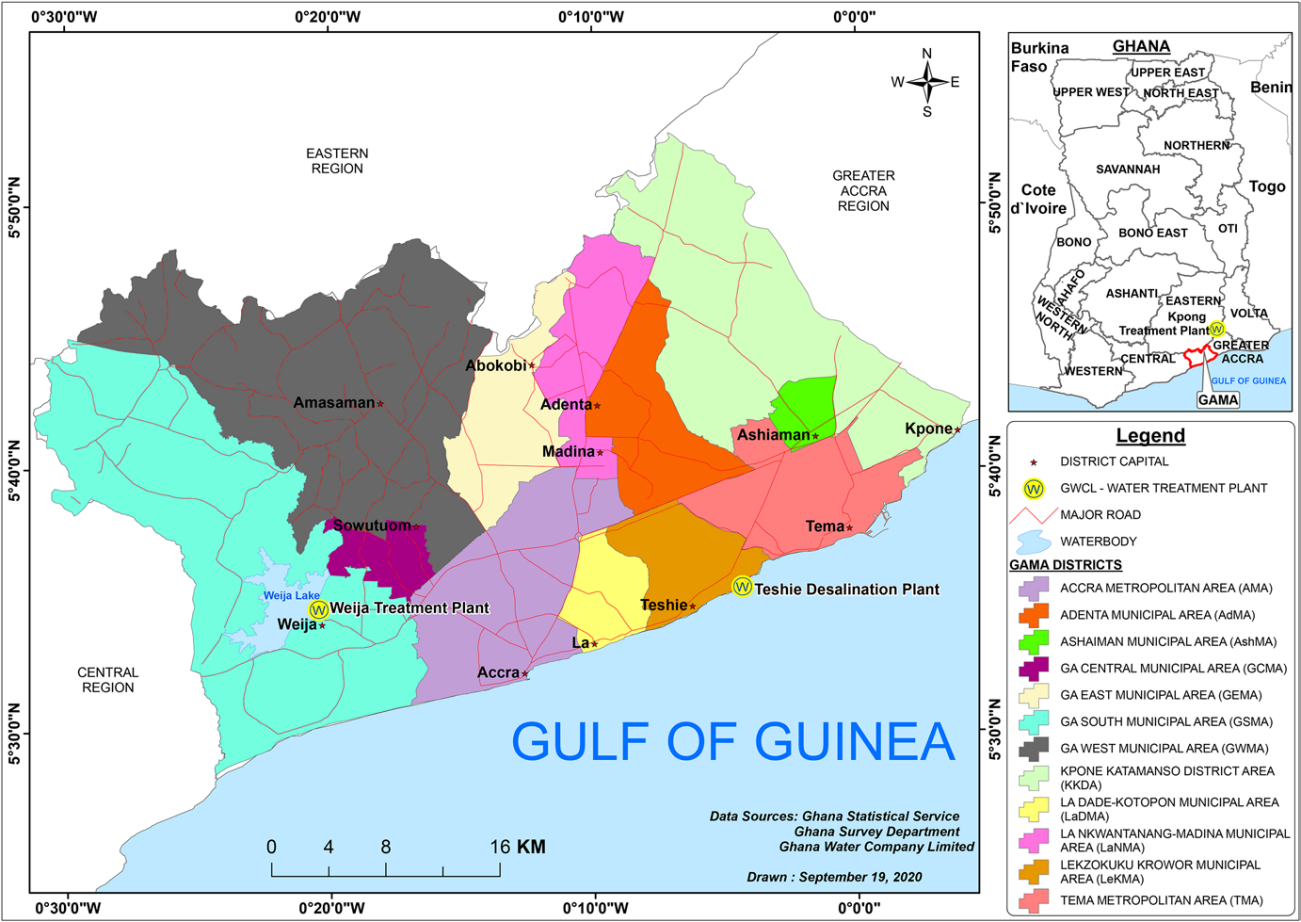
Sub-Administrative demarcation of GAMA as at the 2010 PHC

The study was undertaken in the Greater Accra Metropolitan Area (GAMA). Administratively, GAMA has undergone various transformations and fragmentation. Table 2 shows that GAMA was formally divided into three main districts: Accra Metropolitan Area (AMA), Tema Municipal Area (TMA), and the Ga District (GSS, 2005). In 2004, these local government areas increased from three to four and later doubled in 2008 in response to mainly the phenomenon of urban growth and sprawl (Owusu, 2015). As of 2012, GAMA comprised of 12 metropolitan, municipal, and district assemblies (MMDAs) (Fig. 1, Table 2). For this analysis, we use the 12 MMDAs present from 2012. Presently, this contiguous built-up metropolitan area has 25 MMDAs (Ghana-Districts, 2020) with estimated projection over 4.7 million residents (GSS, 2020). These MMDAs are where policy decisions are implemented.

**Table 2:** Population and EA distribution in GAMA by administrative units 2012

Administrative sub-division (1988)		Total population 2000		Administrative sub-division (2004)		Administrative sub-division (2008)	Administrative sub-division (2012)	Total population 2010	Number of EAs in 2010
*Accra Metropolitan Area*		*1,658,937*		*AMA*		*AMA*	*AMA*	*1,665,086*	*1929*
							*LaDMA*	*183,528*	*207*
						*LeKMA*	*LeKMA*	*227,932*	*288*
*Tema Municipal Area*		*506,400*		TMA		TMA	TMA	292,773	379
							KKDA	109,864	132
						AshMA	AshMA	190,972	260
						AdMA	AdMA	78,215	261
*Ga District*		*550,468*		*GEMA*		*GEMA*	*LaNMA*	*111,926*	*219*
							*GEMA*	*147,742*	*228*
				*GWMA*		*GWMA*	*GWMA*	*219,788*	*363*
							*GCMA*	*117,220*	*172*
						*GSMA*	*GSMA*	*411,377*	*581*

Sources: Derived from Population Census Reports for 2000 and 2010 (GSS, 2005, 2014a, b, c, d, e, f, g, h, i, j, k, l, m; Owusu, 2015)

Of the 12 administrative districts, the AMA has the largest population of over 1.6 million people while Adenta Municipal Area recorded the lowest population of about 78,200 (Table 2). In 2010, GAMA recorded a population of close to 3.8 million inhabitants occupying 5019 enumeration areas (EAs). The AMA has the most EAs, followed by the Ga South Municipal Area, then by the TMA while Kpone Katamanso District is observed as having the least EAs coverage (See Table 2; Fig. 3).

**Fig. 2:**
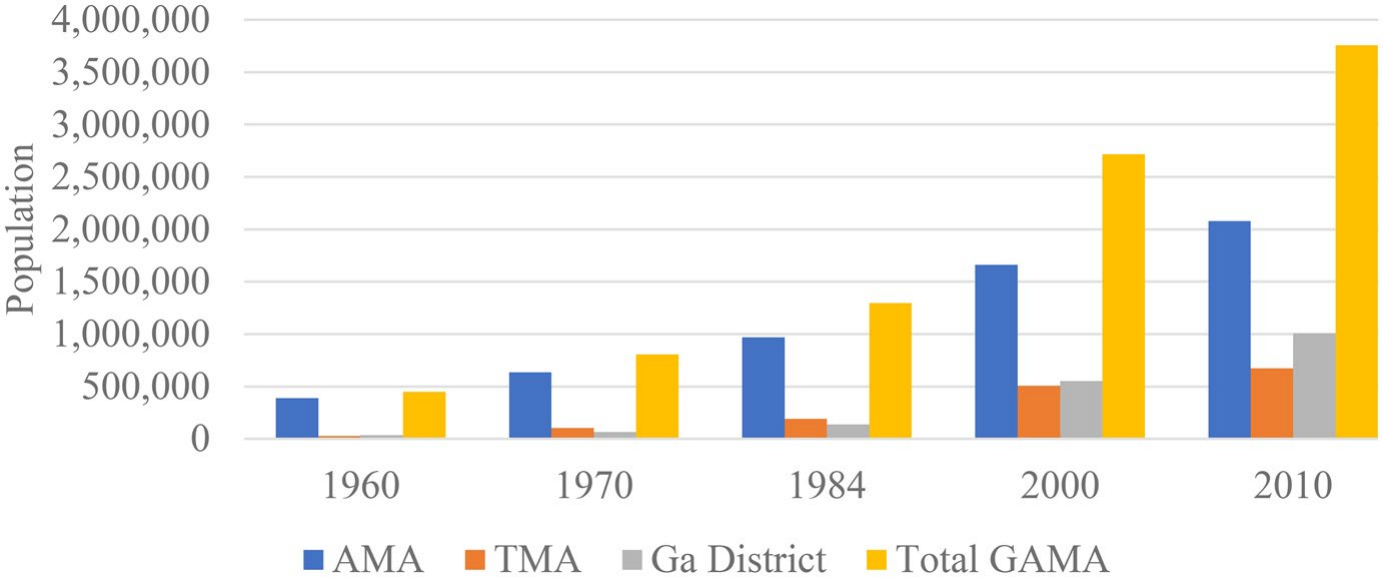
Population of GAMA from 1960 to 2010 (source: Derived from Ghana Statistical Service, Population Census Reports for 1960, 1970, 1984, 2000, 2010 censuses) (see Census Office, 1960, 1975; GSS, 1987, 2005, 2012; Yankson & Bertrand, 2012)

Based on census reports, the Ga District experienced the highest growth rate of about 58% annually from 1960 to 2010, followed by the TMA (47.5%) with AMA experiencing the least growth rate of about 9% even though is the most populated region. The whole of GAMA, notwithstanding, grew at about 15% annually from 1960 to 2010 (see Fig. 2).

Urbanization and urban expansion are central to our understanding of variation in water demand and use in GAMA. Accra Metropolitan Area (AMA)’s population growth in recent decades is largely due to its growing position as an industrial, administrative, and commercial center (Agyei-Mensah & Wrigley-Asante, 2014). As the seat of government, the high cost of land and limited residential spaces have push migrants and low-income residents into the creation of slums and squatter settlements which often lack essential services such as water and sanitation (Yankson & Bertrand, 2012). Although the population of Tema Metropolitan Area (a planned industrial hub) has increased beyond the core communities since independence, housing developments and water provision has been undertaken within the context of planning before development. The Ga Districts have experienced perhaps the most significant population growth over the years because of the availability of undeveloped land and congestion in AMA. This growth constrains access to water provision (Owusu, 2015; Yankson & Bertrand, 2012).

In terms of water supply, GAMA is served mainly with surface water from two major water treatment plants: the Kpong and the Weija Treatment Plants. About 8% of water also comes from the Teshie Desalination Plant if it is in operation. The Weija Treatment Plant and the Teshie Desalination Treatment Plant are located within GAMA (Fig. 1) while the Kpong Treatment Plant is located 54 km North-East of Tema (GWCL, 2020a, c). These plants are under the management of the GWCL—the main urban water utility company in Ghana (GWCL, 2020b). For the purposes of their operations, GAMA is divided into three regions: Accra East, Accra West, and Tema (GWCL, 2018).

### Research design and analytical framework

**Fig. 3:**
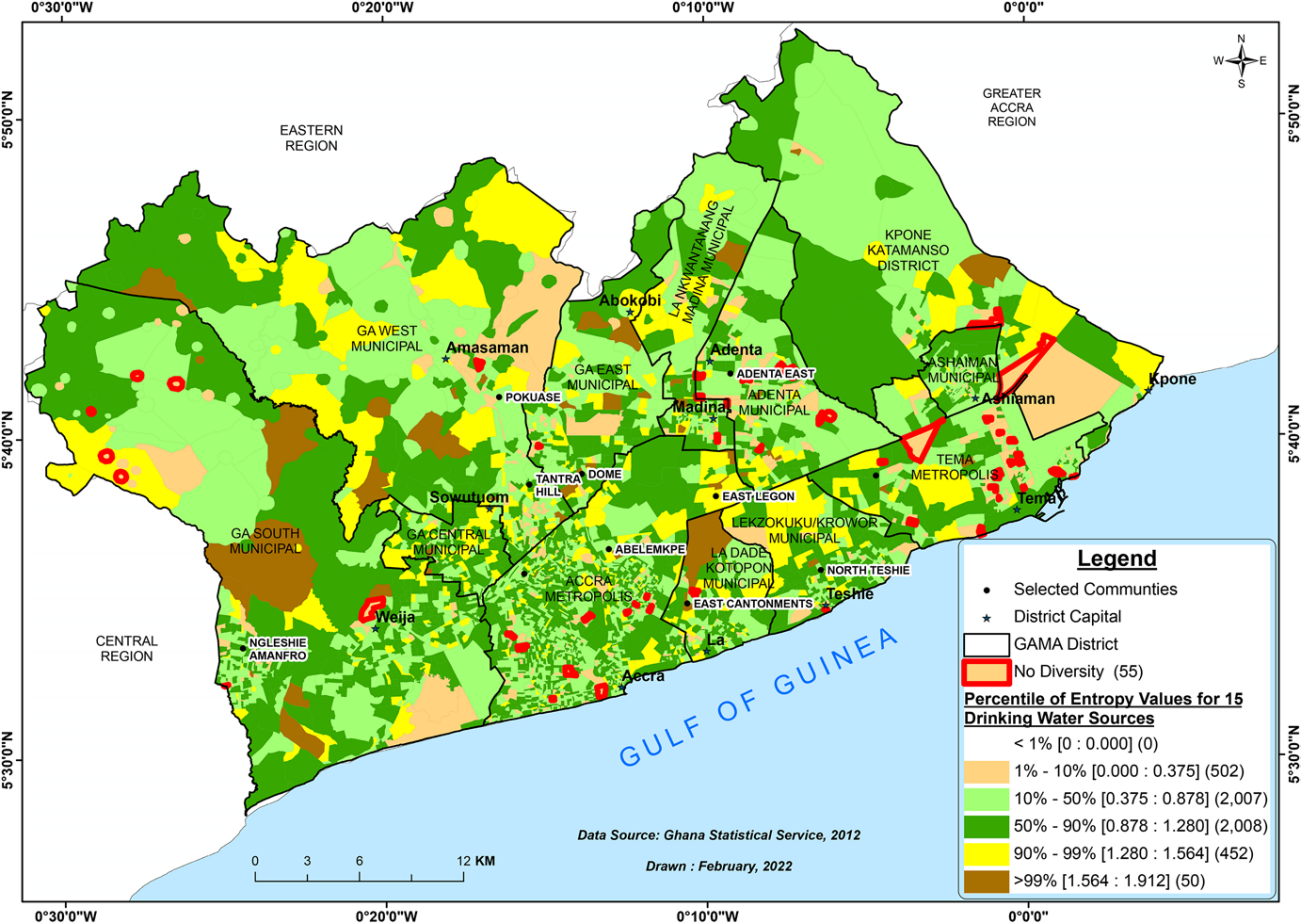
Diversity of the various drinking water sources at the EA level

This analysis relies on the full microdata from the 2010 Population and Housing Census (PHC) from the Ghana Statistical Service of Ghana (GSS). Using this data, we constructed an enumeration area (EA)-level dataset to examine spatial heterogeneities in drinking water sources in GAMA. The datasets are geo-referenced to the EA level, of which all the 5019 EAs in the GAMA were used for our analysis (see Table 2, Fig. 3). An EA, which has a mean area of 0.301 km^2^, is an area of land demarcated in order to enumerate houses, structures, and households during the Census (GSS, 2010a). These small geographic units represent sub-communities, allowing us to examine variations within communities that are typically subsumed into larger districts. Using this dataset, we first constructed an entropy index that provides a continuous measure of heterogeneity of drinking water sources in each EA. Second, we grouped the drinking water sources of each household into four categories: piped water sources, vendor sources, other improved sources, and other unimproved source (see Table 1) to describe the similarities and differences of drinking water sources of the 12 districts. Furthermore, we estimated the income inequalities of neighborhoods across GAMA. Lastly, we estimated associations between the diversity of drinking water sources and neighborhood income segregation. With these indicators, we examine how variability in drinking water and segregation are correlated as well as how they are spatially related using a bivariate local indicator of spatial association (BiLISA) approach along with the most common drinking water types**.**

### Categorizing household sources of drinking water

We used all the water sources listed in the census. There are two questions on water sources included in the household module of the census. Our analysis relied mainly on answers to the question *what is the main source of drinking water for the household*? The 2010 PHC questionnaire survey lists 15 sources of water sources (GSS, 2010b, see also Table 1).

We considered the classification scheme used by the JMP of the WHO and UNICEF. The scheme is categorized into the improved and unimproved sources of drinking water types. The improved sources include pipe-borne inside dwelling, pipe-borne outside dwelling, public tap/standpipe, borehole/pump/tube well, protected well, rainwater, protected spring, bottled water, sachet water, and tanker supply/vendor provided. Non-improved sources consist of the unprotected well, unprotected spring, river/stream, and dugout/pond/lake/dam/canal (GSS, 2010b; UNICEF and WHO, 2019). The JMP further classifies improved household drinking water as being limited, basic, or safely managed services based on accessibility, availability, and quality criteria. However, the 2010 PHC lacks enough data for the estimation of these services (UNICEF and WHO, 2019).

### Estimation of Shannon entropy index

We used the Shannon entropy index in order to offer greater insight into the spatial aspects of household drinking water inequality. This index is a continuous measure of the degree of variation that reveal the different dimensions in water insecurity and the multiple strategies households use to obtain drinking water. Therefore, we calculated the entropy index for each EA based on the diverse household drinking water sources. This index provides a quantitative measure (ranging from 0 to 1.912 in Fig. 3) of the level of heterogeneity in drinking water sources among households in each EA. The Shannon entropy index was initially applied spatially in the context of segregation by Theil and Finizza (1971) and is defined in White (1986) as:

$$h_i-{\textstyle\sum_{j=1}^k}p_{ij}ln{(p}_{ij})$$

where *h* is the entropy at EA *i*, *k* is the number of individual drinking water sources, and ${p}_{ij}$ is the proportion of a *j*th drinking water source in EA *i* (${p}_{ij}$=${n}_{ij}/{n}_{i}$, ${n}_{ij}$ is the count of *jth* drinking water source in an EA *i*, and ${n}_{i}$ is the total count of all drinking water sources in an EA *i*) (Bandt, 2020; White, 1986). The higher the value of ${h}_{i}$, the higher the diversity of drinking water sources. Lower values indicate lower diversity while a value of zero indicates that the community has only one drinking water source (Iceland, 2004; Reardon & Firebaugh, 2002). High diversity is associated with largely vendor water sources particularly sachet water while low diversity is associated with mainly the piped water sources.

### Estimation of income inequality

We also considered how spatial income inequality varies across and within the 12 districts and is associated with community water source variability. While wealth is considered a predictor of access to improved water, neighborhood context may disrupt households’ ability to access improved water sources. Thus, to get a better idea of how households navigate a complex water landscape, we examine how community economic status impacts these strategies.

We used the Index of Concentration at the Extremes^5^ (ICE) to classify EA-level socio-economic status (i.e. low- and high-income areas), by measuring the degree to which GAMA’s population is concentrated into extremes of wealth and poverty. Consumption data in Ghana is available in the Ghana Living Standard Survey 6 (GLSS 6),^6^ but it can only be disaggregated to the district-level. As part of our effort to produce an EA-level dataset, we employed a small area estimation procedure to produce consumption indicators at the EA-level (Elbers et al., 2003). This method borrows strength from survey information by fitting a linear mixed model with random effects at the area-level to GLSS data and uses parameter estimates to predict consumption for all census households (Molina & Rao, 2010; Rodas et al., 2021). To develop the ICE metric, we grouped households by their relative position in the GAMA consumption distribution; households in the bottom 20% are considered low-income, while those in the top 20% are considered high income. ICE is defined as:

$${ICE}_{i}=\left({A}_{i}- {P}_{i}\right)/{T}_{i}$$

where, for EA *i*, ${A}_{i}$ is the number of people in high-income households; ${P}_{i}$ is the number of people in low-income households; and ${T}_{i}$ is the EA-level population. ICE ranges from − 1 to 1, with negative values indicating concentration of poverty, while values closer to 1 indicate clustering of affluence (Krieger et al., 2016). EAs were then classified based on their ICE value; the bottom 20% of EAs were classified as low income or deprived while the top 20% were classified as high income or privileged (Chambers et al., 2019; Krieger et al., 2016). This metric allows us to examine the extremes of GAMA’s consumption distribution in one metric, and allows us to identify the most polarized EAs.

### Spatial analysis of drinking water source and neighborhood income

To identify where the associations between drinking water and neighborhood wealth emerge within GAMA, we applied bivariate local indicators of spatial association^7^ (BiLISA) to two continuous variables representing diversity in drinking water sources (Table 2) and socio-economic status for each EA, the water entropy index, and ICE (Anselin et al., 2002). It identifies the relationship between the value of one variable at location *i (x*_*i*_*)* and the average of neighboring values for a second variable (i.e., spatial lag of *y*_*i*_). The BiLISA statistic is defined as:

$${I}_{i}^{B}=c{x}_{i}\sum_{j}{w}_{ij}{y}_{j}$$

where *c* is a constant scaling factor, and ${w}_{ij}$ are the elements of the spatial weights matrix. We define ${w}_{ij}$ as a second-order Queen’s spatial weights matrix. The resulting bivariate cluster map shows the spatial correlation between water inequality and income status.

Interpretation of this analysis is based on the idea that households in communities with different levels of wealth use different strategies to access improved water. Just because a household is in an affluent neighborhood does not mean that they have access to improved water; thus, the entropy index becomes a signal that households face barriers to accessing improved water beyond limited household budgets constraining consumption choices**.** Communities with high variability near areas of concentrated affluence indicate that households must employ a variety of strategies to obtain water and it is a signal that the water landscape is not as safe as it should be in GAMA. On the other hand, wealthy communities with low variability may indicate that these areas have consistent and improved water sources since households that can afford them are not actively relying on alternative strategies to obtain water.

By the same token, poor communities will have less ability to make room in their household budgets to obtain improved water and are likely to have fewer options. Poorer communities with high variability may suggest that these communities have physical access to multiple sources, but some households cannot afford improved water and must employ alternative strategies. Poorer neighborhoods with little variability could signal that there are no alternatives to poor quality water, or that the community has access to improved drinking water and has no need to look elsewhere. Thus, we use the BiLISA clusters to identify communities that face barriers to accessing improved water.

## Results

### Geographical variations/distribution in drinking water sources in GAMA

Generally, the most prevalent drinking water source among all the diverse sources of water is the piped water sources (i.e., pipe-borne inside dwelling (28.2%), pipe-borne outside dwelling (27.2%), and public tap/stand pipe (8.8%)) and this accounted for 64.2% out of the 980,127 households in GAMA. This was followed by the 28.9% of sachet water prevalence (see Tables 1 and 3). On the other hand, tanker supply/vendor provided, borehole/pump/tube well, and bottled water sources constituted 2.8%, 1.5%, and 1.1% respectively. However, eight different drinking water sources were all less than 1%. They include protected well (0.9%), rain water (0.2%), protected spring (0.3%), unprotected well (0.1%), unprotected spring (0.02%), river/stream (0.2%), dam/pond/lake/dugout/canal (0.1%), and other (0.1%). In effect, the unprotected spring was the least accessed drinking water source in all of the GAMA region. In terms of improved sources of drinking water, over 99% (*n* = 974,724) of households accessed an improved source of drinking water in this metropolitan region (see also Tables 1 and 3).

### Diversity of drinking water sources in GAMA

Figure 3 shows the Shannon entropy values of all the EAs within GAMA (*n* = 5019). This measure indicates the diversity of the various drinking water sources at the EA level across this urban agglomeration. This diversity helps us to understand the different choices households make in accessing multiple drinking water sources. The analysis reveals that 55 EAs within GAMA recorded a value of zero (i.e., EAs highlighted in red) meaning that these EAs had no diversity and only accessed a particular type of drinking water source. These no diversity EAs mostly utilized improved water sources (96.4%) with 58% of those sources being pipe-borne inside the dwelling and this can found mainly in the AMA and TMA. Other EAs that consume only sachet water and borehole/pump/tube well sources are seen mainly in the Adentan and Ga South Municipalities, respectively. The remaining 3.6% of the no diversity EAs utilized only unprotected well. The highlighted EAs are included in the 10th percentile, demarcating a group of EAs that have the lowest diversity (*n* = 502). On the other hand, fewer EAs (*n* = 50) registered the most diversity (i.e., > 99th percentile). Households in these fewer EAs are observed to have depended on any of the 15 drinking water sources.

However, the majority of EAs show middling levels of diversity (*n* = 4467) accessing mostly the improved sources of drinking water (see Fig. 3). Thus, the entropy analysis shows that most places in GAMA fall somewhere in the middle of the distribution.

### Neighborhood wealth and drinking water sources

**Fig. 4:**
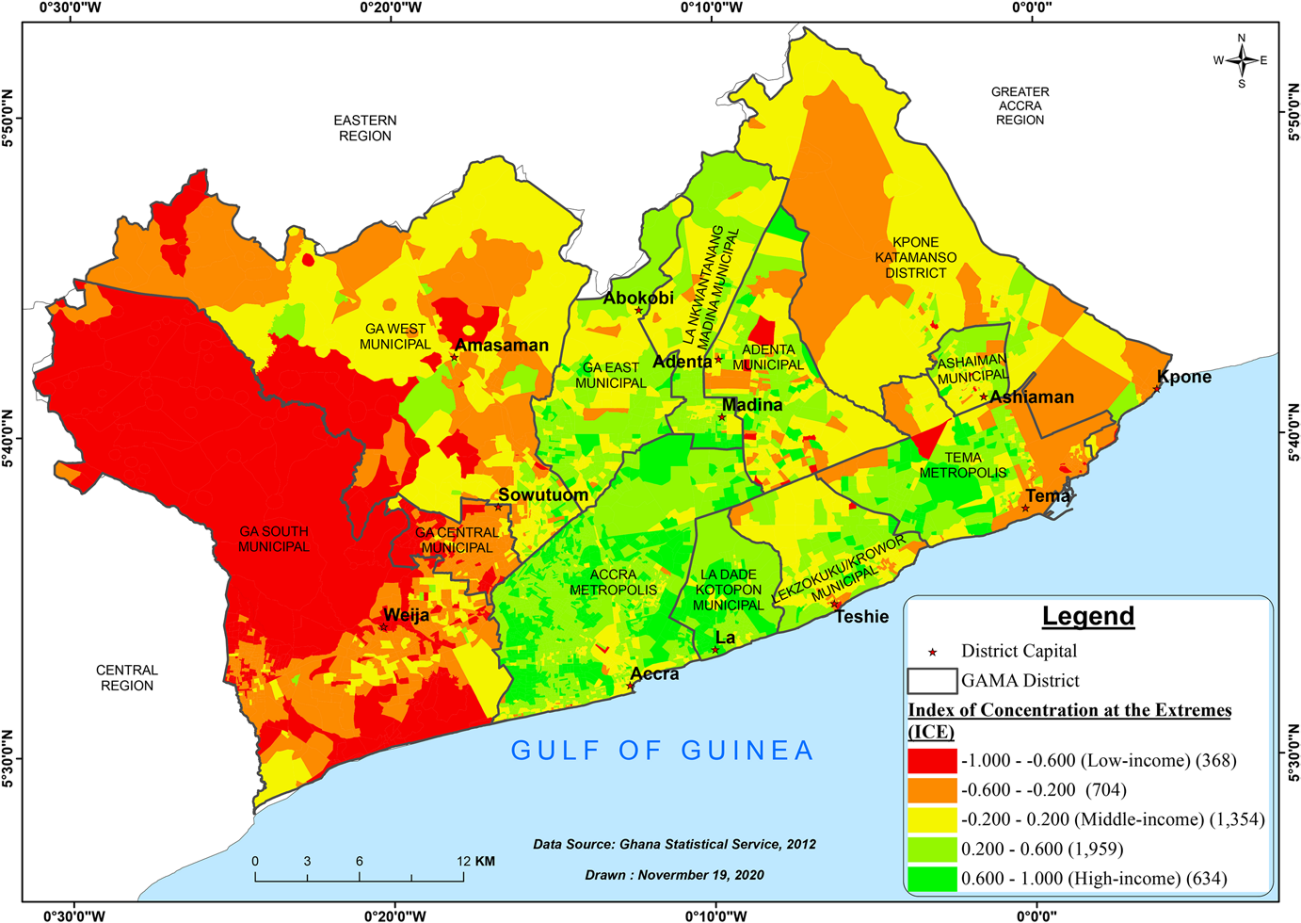
Neighborhood wealth status

Figure 4 shows the wealth indices of the EAs across the various districts of GAMA based on the Index of Concentration at the Extremes. These indices describe economic segregation in GAMA. Several wealthy neighborhoods can be found in the Accra Metropolitan Area, La Dade-Kotopon Municipal Area, Tema Metropolitan Area, La Nkwantanang-Madina Municipal Area, and the Ga East Municipal Area. Poorer areas can be seen more in districts such as the Ga South Municipal and Ga West Municipal Areas. Although the majority of EAs in GAMA fall under the middle-income bracket, the wealth map largely shows significant differences among the various districts.

**Fig. 5:**
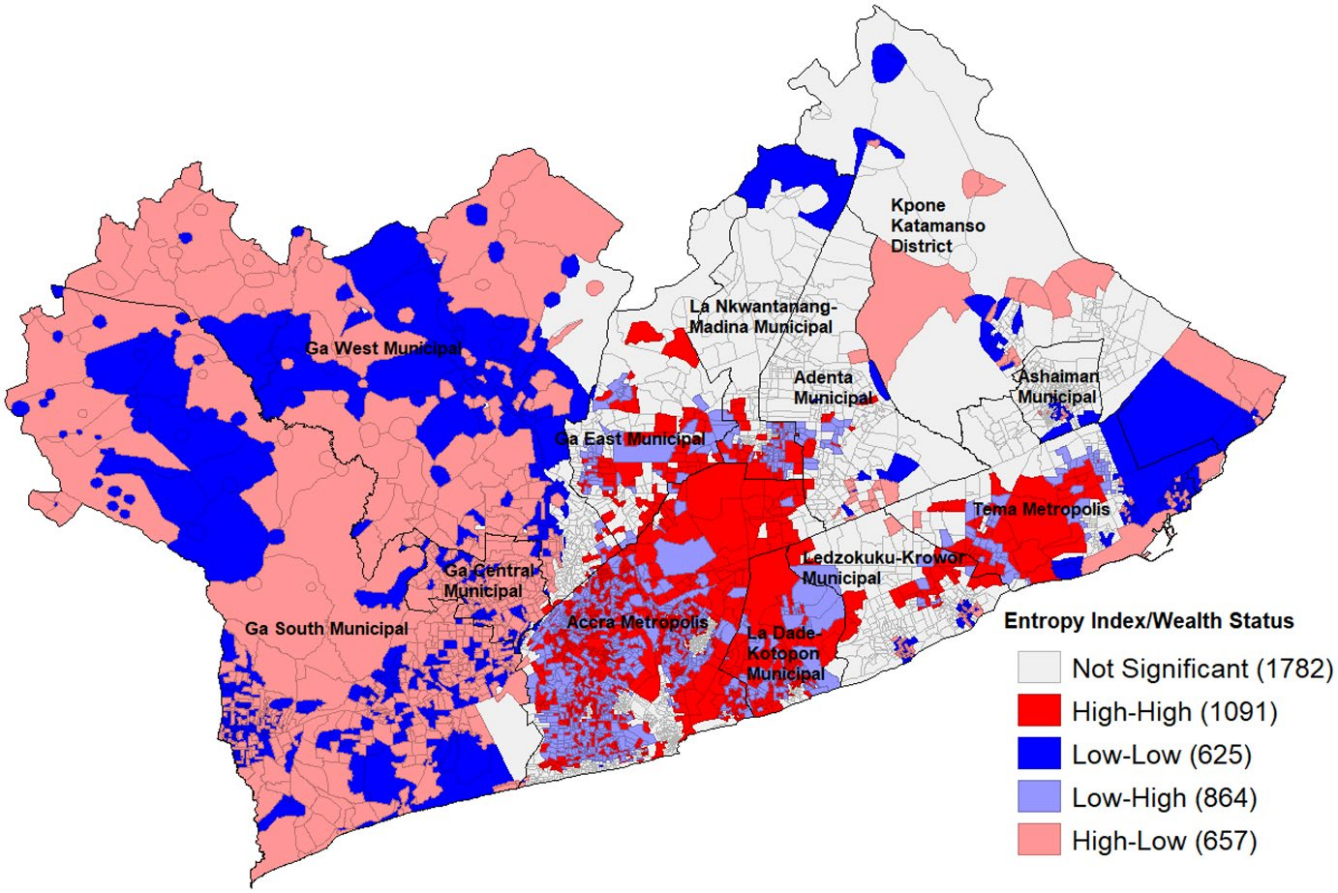
Diversity in drinking water sources versus income status

Furthermore, we examined how the diversity of the water landscape is affected by socio-economic status, so we correlated the entropy index with the ICE to identify the areas of GAMA that are marked by concentrated affluence and concentrated poverty. Figure 5 presents the results from the cluster detection analysis from the diversity of various drinking water sources against low- and high-income residential areas, showing the spatial association between diverse drinking water sources and wealth status. This figure shows a situation where clusters of high diversity in drinking water sources in high-income residential areas are mainly found in the Accra and Tema Metropolises along with La Dade-Kotopon and Ga East Municipalities. High-high clusters can also be observed within the Ledzokuku-Krowor Municipality as well as the lower parts of La Nkwantanang-Madina Municipality close to the Accra Metropolis boundary. It is also important to note that the abovementioned districts which are more urbanized also exhibited low–high clusters meaning that there are also high-income residential areas with low diversity in access to drinking water sources (Fig. 5).

The above scenario is contrasted with low diversity in low-income areas which can clearly be seen in the industrial zones between the Tema Metropolis and the Kpone Katamanso District. These are also observed in some significant sections of the Ga West, Ga Central, and Ga South Municipalities. On the other hand, high-low clusters represent high diversity in drinking water sources in low-income areas and can be visibly seen in the large portions of the Ga West, Ga Central, and Ga South Municipalities alongside isolated sections of the Kpone Katamanso District which are all largely within the peri-urban areas of GAMA.

We were also interested in the most common types of drinking water against the backdrop of entropy and wealth status (Table 3). From Table 3, the residents of high diversity drinking water sources in high-income areas mostly consume sachet water (32.6%), pipe-borne inside dwelling (27.2%), pipe-borne outside dwelling (25.7%), public tap/stand pipe (9.3%), and then bottled water (2.1%). Also, the high diversity in low-income areas patronized sachet water (32.7%), pipe-borne outside dwelling (27.0%), pipe-borne inside dwelling (16.2%), public tap/standpipe (10.0%),and tanker services (4.8%) as their most common types of drinking water. For the low diversity in low-income neighborhoods, the most common drinking water sources were the pipe-borne outside dwelling (38.5%), sachet water (21.1%), pipe-borne inside dwelling (18.9%), public tap/standpipe (10.5%), and the tanker services (5.3%). In contrast, the low diversity in high-income areas commonly consume from the pipe-borne inside dwelling (44.9%), sachet water (24.6%), pipe-borne outside dwelling (23.5%), public tap/stand pipe (4.0%), and tanker services (1.5%). Irrespective of residents’ socio-economic status, sachet water usage is largely associated with areas of highly diverse drinking water sources making it ubiquitous while the pipe-borne water can normally be found in areas of low diversity.

**Table 3:** Entropy/wealth status and most common drinking water types (in percentages)

	Not significant	High-high	Low-low	Low–high	High-low	GAMA total
Pipe-borne inside dwelling	24.5	27.2	18.9	44.9	16.2	27.2
Pipe-borne outside dwelling	29.8	25.7	38.5	23.5	27.0	28.2
Public tap/standpipe	10.1	9.3	10.5	4.0	10.0	8.8
Bottled water	0.9	2.1	0.2	0.6	0.9	1.1
Sachet water	29.1	32.6	21.1	24.6	32.7	28.9
Tanker supply/vendor provided	3.2	1.2	5.3	1.5	4.8	2.8
Borehole/pump/tube well	1.3	0.7	3.1	0.4	4.1	1.5
Protected well	0.4	0.4	0.6	0.1	1.3	0.5
Rain water	0.1	0.1	0.2	0.0	0.6	0.2
Protected spring	0.3	0.4	0.3	0.3	0.4	0.3
Unprotected well	0.0	0.1	0.3	0.0	0.3	0.1
Unprotected spring	0.0	0.0	0.1	0.0	0.1	0.0
River/stream	0.1	0.1	0.5	0.0	1.0	0.2
Dugout/pond/lake/dam/canal	0.0	0.0	0.3	0.0	0.5	0.1
Other (specify)	0.1	0.2	0.0	0.1	0.1	0.1

## Discussion

This study sought to examine spatial heterogeneity in drinking water sources and neighborhood income in GAMA. While previous research has explored drinking water sources based on sample survey data of selected neighborhoods of GAMA (Benneh et al., 1993; Songsore & McGranahan, 1993, 1998; Stoler et al., 2013), to the best of our knowledge, ours is the first to analyze drinking water sources based on census mapping at the enumeration area level. One important advantage of a census is its comprehensive coverage which enables the researcher to analyze data at localized settings. The study adds to the growing body of literature notably among geographers who have explored inequality using census data at different spatial scales to study environmental and demographic issues (Arku et al., 2016; Cutter & Finch, 2008; Weeks et al., 2010).

Using the Shannon entropy index and the index of concentration at the extremes, we have showed considerable spatial diversity and accompanying inequalities in drinking water sources not evidenced in previous studies. There is evidence of high socio-economic areas with high diversity in drinking water sources as well as areas of high socio-economic status with low diversity in drinking water sources. Likewise, we also find areas of low socio-economic status with high diversity in drinking water as well as areas of low socio-economic status with low diversity in drinking water sources.

By complementing the entropy/wealth measure with the most common types of water used, we have been able to provide evidence of how the meaning of heterogeneity in water sources differ in wealthy and poor EAs (see Table 3). Thus, heterogeneity in drinking water sources in GAMA is good as evidenced by the dominance of piped water sources which are at the top of the JMP ladder and are publicly managed, improved, and affordable. In addition, it can be argued that a significant proportion of the population also depend on vendor sources especially sachet water which suggests a heavier burden in terms of costs placed on consumers. Thus, Table 3 provides compelling evidence that there are different strategies within each BiLISA category for accessing drinking water in GAMA and thus heterogeneity is neither good nor bad.

Given the segregated nature of Accra’s neighborhoods based on socio-economic status (Agyei-Mensah & Owusu, 2010), both the rich and the poor adopt multiple strategies in looking for drinking water. For the urban poor who may not have regular access or connection to pipe borne water, the alternatives available are sachet water, bottled water, water tankers, and in some instances unprotected well and rainwater. For the rich, many of whom may have access to pipe borne water inside dwelling; the use of multiple sources such as sachet water and bottled water may reflect the perception of water quality and irregular flow of pipe borne water as well as water rationing. Perceived and actual quality of water from Ghana’s piped system is highly variable as noted by Morinville (2017). Unfortunately, census data does not provide questions on water quality. The use of sachet water among many residents of GAMA (see Table 2 and Fig. 3) reinforces Moulds et al. (2022) assertion that sachet water has become ubiquitous in urban Ghana.

With regard to the factors driving these patterns, our results suggest multiple factors related to challenges with the municipal water delivery system and governance, wealth differences, population growth and urban expansion, and the colonial and post-colonial imprint on the urban landscape. The impact of these factors varies significantly based on geographical location. The Accra Metropolitan Area (AMA) is the oldest settlement among the administrative districts of GAMA, and home to the Gas—the indigenous settlers of Accra. Most residents here depend on multiple water sources such as pipe borne water followed by sachet water with some segments of the population depending on bottled water and water tankers. Note that most of the water sources here are improved water sources going by JMP ladder. The drivers of water consumption pattern at AMA can be traced to the colonial and post-colonial era. As noted in the literature on colonial urbanization, Accra’s spatial structure was heavily influenced by the colonialists (Agyei-Mensah & Owusu, 2010; Andersson, 2017; Bohman, 2012; Brand, 1972; Harris, 2021; Songsore et al., 2009). Areas such as Airport Residential Area, Ridges, and Cantonments were provided with well laid out infrastructure in terms of the provision of water services. This has continued into the post-colonial era with the residential facilities being provided for top African civil and public servants and the diplomatic community.

The use of multiple water sources in the indigenous Ga areas such as Jamestown, Ussher Town, Chorkor and Teshie can be traced to the legacies of uneven infrastructure development during the colonial period, intermittent water flows and rationing as well as payment of water bills (see Adams & Vásquez, 2019; Brand, 1972; Harris, 2021; Sualihu et al., 2017). Population growth and urban expansion driven largely by migration has also pushed migrants who come into the city into slums or informal settlements (such as Nima, Maamobi and Sabon Zongo) and peri urban areas such as Abokobi, Amasaman, and Kpone with limited supply of pipe-borne water services. EAs in these low-income areas depend on multiple sources of water outside the main formal system. Even where there are water connections, the taps do not flow regularly due to the rationing system as well as lack of maintenance and aging water pipes and lines (Dapaah & Harris, 2017; Harris, 2021). As a result, they resort to other multiple sources such sachet water as well as tanker water sources. In addition, it is important to note that the La Dade-Kotopon Municipal Area (LaDMA) as well as the Ledzokuku-Krowor Municipal Area (LeKMA) share very similar characteristics since they were recently carved out of the AMA (see Table 2).

Compared to AMA, the situation in the Tema Metropolitan Area (TMA) looks different. Here, most of the population depend on piped water sources. In addition, segments of the population also consume sachet and bottled drinking water. As noted earlier, the case of Tema Metropolis can be traced to investments in the provision of water infrastructure that accompanied the development of TMA as a planned industrial city by the state after independence in 1957 (Kirchherr, 1968; Songsore et al., 2009). These developments were driven by the establishment of the Tema Development Corporation and subsequently the growth of private estate developers that led to the expansion of the traditional core residential communities (communities 1–12) to the current 25 communities. It is noteworthy that all these communities obtain their water through the Ghana Water Company Limited. TMA’s situation has largely been driven by *planning before development* and its ability to control population growth and urban expansion by the provision of planned residential communities. The use of sachet and bottle water by segments of the population may reflect on some of the challenges in the piped borne supply system relating to rationing and intermittent flows (Asante-Wusu & Yeboah, 2020) as well as the quality of water coming from the taps**.**

A different pattern emerges in the Adentan Municipality where residents depend largely on sachet water, followed by pipe borne, tankers, and bottled water. Compared with AMA and TMA, this area is relatively new with most development occurring after the 1980s. Many housing developments in the Adentan area were constructed without formal planning in terms of the provision of water infrastructure as well as electricity and tarred roads, and most settlements extend beyond the limits of the two main water delivery systems. The use of vendor water sources such as sachet water and tanker services for drinking in many households in the Adentan Municipality reflects in part the unavailability of piped borne water systems in some homes and where they exist the intermittent flow/rationing (Alba et al., 2019; Bartels et al., 2018; Dapaah & Harris, 2017; Tutu & Stoler, 2016). Thus, most households resort to sachet water, bottled water, and tanker services. Population growth and lack of access to land in AMA have pushed many people to the outskirts including Adentan Municipality. In most cases, residential facilities have been put up without the provision of water systems. Thus, this situation reflects largely the unavailability of pipe-borne water systems creating a demand for sachet water and tanker service deliveries. Thus, unlike TMA, it reflects a *development before planning* approach.

Unlike AMA, LaDMA, LeKMA, TMA, and the Adentan Municipality described above, the Ga District, namely the Ga East, Ga West, Ga Central, and Ga South Municipalities as well as the La Nkwantanang-Madina Municipality, depend on multiple water sources. Sachet water is the dominant water use except for the Ga South which depends largely on piped borne water probably due to its closeness to the Weija Treatment Plant. Other water sources here are piped water, tankers, bottled water, and some unimproved sources. Two major factors are driving water consumption here: population growth and unavailability of water infrastructure. For example, the Ga District recorded the highest population growth rate of nearly 60 percent per annum between 1960 and 2010 among the 12 districts of GAMA (Fig. 2). Most of this increase is due to lack of land in Accra and the movement of the population to the outskirts of the main city (Yankson & Bertrand, 2012). These movements to the outskirts are usually not accompanied by expansion in water infrastructure. Thus, there is a disconnect between population increase and water infrastructure in the Ga District (see also Mosello, 2017) as well as the other districts.

Our study has some limitations to consider for future work. The census data used here is the most recently available one for Ghana but still a decade old, with a 2021 census currently being processed (delayed from 2020 due to the Covid-19 pandemic). Nevertheless, our study provides a valuable baseline against which to evaluate the data from this census, to identify key temporal trends, and use as benchmarks to measure the success of policies towards universal access. It should also be noted that our focus was on drinking water sources at the enumeration and household levels. As such, people’s water sources at work and school were not captured here as they are not represented in the census.

The analysis was also based essentially on census data and did not involve interviews of households. Thus, actual water users were not interviewed**.** With respect to water quality, some studies such as one by Sunkari and Danladi (2016) in the Accra Metropolis on bottled drinking water indicate that the trace elements pose no risks as they are below the WHO and the Ghana Standard Authority’s guidelines. Another study in the Ga West Municipality on borehole water also showed that the physico-chemical properties for drinking water are within the required limits of the WHO except for total dissolved solids (TDS), total hardness, sodium (Na^+^), and chlorine (Cl^−^) which could be as a result of solid waste leachate and marine water intrusion (Sunkari et al., 2019).

Despite these limitations, the study provides a comprehensive portrait of the different drinking water sources in localized setting as well as the drivers of water consumption.

## Conclusions

In this article, we have examined the spatial heterogeneity in drinking water sources within the neighborhoods of GAMA, and how they differ by neighborhood wealth status. Prior literature on this subject in GAMA have mostly utilized sample survey data. Our analysis relies on census data that provides a comprehensive coverage of sources of drinking water at the enumeration area level. Based on the Shannon entropy index and the ICE, we have shown considerable spatial heterogeneity and inequality in drinking water sources not evidenced in previous studies. By complementing the entropy/wealth status with the most common water sources, we conclude that heterogeneity is neither good nor bad judging by the dominance of both piped water sources and vendor sources such as sachet water.

The drivers of these heterogeneities are also not uniform and relate to a disconnect between population/urban territory growth and the provision of pipe borne water infrastructure. Other factors relate to the segregated nature of the urban colonial and post-colonial structure, and challenges faced by municipal authorities in their quest to provide adequate water services to the population. Poverty and wealth also shape the choices people make with respect to sources of drinking water. Of course, there are also issues with the quality of pipe borne water, intermittent flow, rationing, and maintenance.

Taken together, our results suggest that GAMA’s water system is not oriented towards equity and that most residents adopt multiple strategies in their quest for household drinking water. It is also clear that some of housing developments in GAMA such as parts of the Ga District were not preceded by formal planning in terms of the provision of water infrastructure. This contrasts sharply with TMA which was a planned industrial city with provision for water infrastructure. For policy purposes, it is important that policy makers especially officials of GAMA, GWCL, and other agencies in water resources and infrastructure planning, or other anti-poverty initiatives in GAMA, recognize and understand the dramatic spatial heterogeneity of drinking water sources in GAMA. This is important because it is an indicator of profound inequality, and since water access is widely recognized policy priority per the SDG—Goal 6 and vital for improving population health and well-being. Thus, interventions should focus on providing piped water sources in areas not covered with water supply as these sources are more affordable, safely managed, and high on the JMP ladder.

## Data access and availability

Enumeration area level data with their respective household drinking water sources are provided as supporting information at http://equitablehealthycities.org/data-download/. Individual level data and shapefiles may be requested from Ghana Statistical Service or at https://statsghana.gov.gh/gssdatadownloadspage.php.

## Footnotes

1 An improved drinking water source is the source that has the potential of delivering safe water by nature of its design and construction (UNICEF & WHO, 2019).

2 A locality or localized setting is defined as a distinct population cluster (also designated as inhabited place, populated center, settlement) which has a name or locally recognized status (GSS, 2010a). Localities are sometimes also referred to as “communities” in this article.

3 A limited drinking water service is an improved source for which collection time exceeds 30 min for a round trip including queuing; A basic drinking water service is an improved source provided the collection time is not more 30 min of round trip including queuing; A safely managed drinking water service is an improved water source located on premises, available when needed and free from fecal and priority chemical contamination (UNICEF & WHO, 2019).

4 An unimproved drinking water service is drinking water from an unprotected dug well or unprotected spring; A surface water drinking service is drinking water directly from a river, dam, lake, pond, stream, canal, or irrigation canal (UNICEF & WHO, 2019).

5 ICE as developed by Massey “measures the spatial social polarizations of both deprived and privileged socio-economic groups simultaneously in one measure” (Chambers et al., 2019; Krieger et al., 2016; Massey, 2001).

6 Consumption in the GLSS 6 is comprised of expenditure categories including Food and Non-Alcoholic Beverages; Alcoholic Beverages, Tobacco and Narcotics; Clothing and Footwear; Housing, Water, Electricity, Gas and Other Fuels; Furnishings, Household Equipment and Routine Maintenance of the Household; Health; Transport; Communications; Recreation and Culture; Education; Hotels, Cafes and Restaurant; and Miscellaneous Goods and Services.

7 We used the open source GeoDa software (Anselin & Li, 2019) to implement this analysis.
